# Supplementary material for: Content-rich biological network constructed by mining PubMed abstracts
Source: BMC Bioinformatics. 2004 Oct 8;5:147. doi: 10.1186/1471-2105-5-147 (PMC528731; doi:10.1186/1471-2105-5-147)
Supplement: Additional File 2 — The original results of the above study (non-essential files are deleted to keep the file size under the limit set by BMC bioinformatics). [file 1471-2105-5-147-S2.bz2 › chilibotAdditionalFile2/dip30/298ID7486683E1289/html/PIK3R1_PDGFRB.html]

 


 **PIK3R1** and **PDGFRB** 
  
Found 21 abstracts in PubMed, retrieved 21.  
 

 What does Google say? 
 PDF only 
| .edu only 

---

**Interactive relationship** (e.g. stimulation, inhibition, etc)

**Neutral relationship**- The distinct tyrosine residue requirements suggest that GAP and PI3K  [ **PIK3R1** ]  bind different regions of the PDGFR  [ **PDGFRB** ] .  Ref: 1375321 Mol Cell Biol, 1992
- Most likely this was due to the presence of a suramin insensitive intracellular PDGFR  [ **PDGFRB** ]  pool that allowed activation of PI3K  [ **PIK3R1** ]  located in intracellular compartments.  Ref: 11473364 J Cell Physiol, 2001
- Taken together, these studies indicate that GAP and PI3K  [ **PIK3R1** ]  bind directly to the PDGFR  [ **PDGFRB** ]  and have discrete binding sites that include portions of the kinase insert domain.  Ref: 1375321 Mol Cell Biol, 1992
- We constructed and characterized a series of PDGFR  [ **PDGFRB** ]  mutants which contain binding sites for PI3K  [ **PIK3R1** ]  as well as one additional protein, either RasGAP, SHP 2, or PLC gamma.  Ref: 8816504 Mol Cell Biol, 1996
- Our previous studies indicated that PI3K  [ **PIK3R1** ]  and PLC gamma were required for relay of the mitogenic signal of beta PDGFR  [ **PDGFRB** ] .  Ref: 7760802 Mol Cell Biol, 1995
- Whereas the PDGFr  [ **PDGFRB** ]  associated with PtdIns 3 kinase, ras GAP, GRB  [ **PIK3R1** ]  2, and phospholipase C gamma, only GRB  [ **PIK3R1** ]  2 and PtdIns 3 kinase associated with IRS 1.  Ref: 7486683 Ann N Y Acad Sci, 1995
- Previous reports have identified three PDGFR  [ **PDGFRB** ]  tyrosine phosphorylation sites in the kinase insert domain that are important for stable association of GAP and PI3K  [ **PIK3R1** ] .  Ref: 7678051 Mol Cell Biol, 1993
- On ligand induced tyrosine phosphorylation, PDGFR  [ **PDGFRB** ]  associated with phospholipase C PLC gamma 1, Ras GTPase activating protein RasGAP, and phosphatidylinositol 3 kinase PI3K  [ **PIK3R1** ]  but not with the Syp growth factor receptor bound protein 2 Son of Sevenless complex.  Ref: 9612285 Am J Physiol, 1998
- Upon binding of platelet derived growth factor PDGF, the PDGF beta receptor PDGFR  [ **PDGFRB** ]  undergoes autophosphorylation on distinct tyrosine residues and binds several SH2 domain containing signal relay enzymes, including phosphatidylinositol 3 kinase PI3K  [ **PIK3R1** ] , phospholipase C gamma PLC gamma, the GTPase activating protein of Ras RasGAP, and the tyrosine phosphatase SHP 2.  Ref: 8816504 Mol Cell Biol, 1996
- In response to binding of platelet derived growth factor PDGF, the PDGF receptor PDGFR  [ **PDGFRB** ]  beta subunit is phosphorylated on tyrosine residues and associates with numerous signal transduction enzymes, including the GTPase activating protein of ras GAP and phosphatidylinositol 3 kinase PI3K  [ **PIK3R1** ] .  Ref: 1375321 Mol Cell Biol, 1992
- Binding of platelet derived growth factor PDGF to the PDGF receptor PDGFR  [ **PDGFRB** ]  beta subunit triggers receptor tyrosine phosphorylation and the stable association of a number of signal transduction molecules, including phospholipase C gamma PLC gamma, the GTPase activating protein of ras GAP, and phosphatidylinositol 3 kinase PI3K  [ **PIK3R1** ] .  Ref: 7678051 Mol Cell Biol, 1993
- Mutagenesis of a tyrosine Y phosphorylation site, Y751, in the PDGFR  [ **PDGFRB** ] , greatly reduces PI3K  [ **PIK3R1** ] , consistent with the possibility that phosphorylation of Y751 signals association of PI3K  [ **PIK3R1** ] .  Ref: 2170111 EMBO J, 1990
- This possibility was also supported by the observation that the antibody to the PDGFR  [ **PDGFRB** ]  kinase insert Y751 region that blocks association of PI3K  [ **PIK3R1** ]  had only a minor effect on the in vitro binding of GAP.  Ref: 1375321 Mol Cell Biol, 1992
- We describe strategies to i target EGFR, its ligand independent variant EGFRvIII, and PDGFR  [ **PDGFRB** ]  on the cell surface, ii inhibit constitutively activate RAS MAPK and PI3K  [ **PIK3R1** ]  Akt signaling pathways, iii target TP53 mutant tumors, and iv block GBM angiogenesis and invasion.  Ref: 12580545 Brain Pathol, 2003
- However, binding of a diphosphotyrosine containing peptide that mimics an activated platelet derived growth factor receptor beta  [ **PDGFRB** ]  induced an increase in the apparent molecular mass of PI3K  [ **PIK3R1** ] .  Ref: 9837914 J Biol Chem, 1998

**Non-interactive relationship** (e.g. studied together, co-existance, homology, etc.)

- Like PI3K  [ **PIK3R1** ] , GAP associates only with receptors that have been permitted to autophosphorylate, and GAP itself does not require tyrosine phosphate in order to stably associate with the phosphorylated PDGFR  [ **PDGFRB** ] .  Ref: 1375321 Mol Cell Biol, 1992
- To test this we have reconstituted the binding of the PDGFR  [ **PDGFRB** ]  beta subunit and PI3K  [ **PIK3R1** ]  in vitro.  Ref: 2170111 EMBO J, 1990
- To investigate the in vivo relevance of this observation, intracellular inositol trisphosphate IP3 generation and calcium release were examined in HepG2 cells expressing a series of PDGFR  [ **PDGFRB** ]  mutants that activate PLCgamma with or without receptor association with PI3K  [ **PIK3R1** ] .  Ref: 9726983 J Biol Chem, 1998
- Thus, activation of PI3K  [ **PIK3R1** ]  requires not only binding of PI3K  [ **PIK3R1** ]  to the tyrosine phosphorylated PDGFR  [ **PDGFRB** ]  but accumulation of GTP bound Ras as well.  Ref: 8816504 Mol Cell Biol, 1996
- Binding is rapid, saturable and requires phosphorylation of the PDGFR  [ **PDGFRB** ]  at Y751, but does not require PDGF dependent phosphorylation of PI3K  [ **PIK3R1** ] .  Ref: 2170111 EMBO J, 1990
- In conclusion, our results demonstrate that Gi enables the PDGFR  [ **PDGFRB** ]  to signal more efficiently to p42 p44 MAPK, and this appears to be achieved through the regulation of c Src and Grb  [ **PIK3R1** ]  2 PI3K  [ **PIK3R1** ] , which are intermediates in the p42 p44 MAPK cascade.  Ref: 9882612 Biochem J, 1999
- The in vitro and in vivo associations of GAP and PI3K  [ **PIK3R1** ]  activity to these PDGFR  [ **PDGFRB** ]  mutants were indistinguishable.  Ref: 1375321 Mol Cell Biol, 1992
- Focusing on the PLC gamma dependent branch of beta PDGFR  [ **PDGFRB** ]  signaling, we constructed a series of mutant beta PDGFRs that contained the binding sites for pairs of the receptor associated proteins PLC gamma and PI3K  [ **PIK3R1** ] , PLC gamma and GAP, or PLC gamma and Syp.  Ref: 7760802 Mol Cell Biol, 1995
- The beta subunit of the platelet derived growth factor receptor PDGFR  [ **PDGFRB** ]  coprecipitates with a phosphatidyl inositol 3 kinase activity PI3K  [ **PIK3R1** ]  following stimulation of cells by PDGF.  Ref: 2170111 EMBO J, 1990
- Moreover, we further demonstrated a requirement for the activation of phosphatidylinositol 3 kinase PI3K  [ **PIK3R1** ]  in this response by using chemical inhibitors of PI3K  [ **PIK3R1** ] , mutant PDGFR  [ **PDGFRB** ] , and dominant negative PI3K  [ **PIK3R1** ] .  Ref: 10197615 Cancer Res, 1999
- In contrast, PDGF BB stimulation triggered PDGFR  [ **PDGFRB** ]  associated PI3K  [ **PIK3R1** ]  activity.  Ref: 9612285 Am J Physiol, 1998
- In addition, highly purified PI3K  [ **PIK3R1** ]  and GAP associated in the absence of other cellular proteins and neither cooperated nor competed with each other s binding to the PDGFR  [ **PDGFRB** ] .  Ref: 1375321 Mol Cell Biol, 1992
- In this study, we have investigated whether PDGF dependent PI3K  [ **PIK3R1** ]  activation is affected by the other proteins that associate with the PDGFR  [ **PDGFRB** ] .  Ref: 8816504 Mol Cell Biol, 1996
